# Shaping of Regional Differences in Oligodendrocyte Dynamics by Regional Heterogeneity of the Pericellular Microenvironment

**DOI:** 10.3389/fncel.2021.721376

**Published:** 2021-10-08

**Authors:** Amin Sherafat, Friederike Pfeiffer, Akiko Nishiyama

**Affiliations:** ^1^Department of Physiology and Neurobiology, University of Connecticut, Storrs, CT, United States; ^2^Department of Neurophysiology, Institute of Physiology, Eberhard Karls University of Tübingen, Tübingen, Germany; ^3^Institute of Systems Genomics, University of Connecticut, Storrs, CT, United States; ^4^The Institute of Brain and Cognitive Sciences, University of Connecticut, Storrs, CT, United States

**Keywords:** myelin, NG2, PDGF, blood vessels, astrocyte, microglia, neuropilin

## Abstract

Oligodendrocyte precursor cells (OPCs) are glial cells that differentiate into mature oligodendrocytes (OLs) to generate new myelin sheaths. While OPCs are distributed uniformly throughout the gray and white matter in the developing and adult brain, those in white matter proliferate and differentiate into oligodendrocytes at a greater rate than those in gray matter. There is currently lack of evidence to suggest that OPCs comprise genetically and transcriptionally distinct subtypes. Rather, the emerging view is that they exist in different cell and functional states, depending on their location and age. Contrary to the normal brain, demyelinated lesions in the gray matter of multiple sclerosis brains contain more OPCs and OLs and are remyelinated more robustly than those in white matter. The differences in the dynamic behavior of OL lineage cells are likely to be influenced by their microenvironment. There are regional differences in astrocytes, microglia, the vasculature, and the composition of the extracellular matrix (ECM). We will discuss how the regional differences in these elements surrounding OPCs might shape their phenotypic variability in normal and demyelinated states.

## Introduction

Oligodendrocyte precursor cells (OPCs), also known as NG2 glia or polydendrocytes, are uniformly distributed throughout the gray and white matter of the adult central nervous system (CNS). They are lineage committed precursor cells whose primary known function is to differentiate into mature oligodendrocytes (OLs) that myelinate axons and enable fast saltatory conduction of action potentials. OPCs express the cell surface proteins NG2 and platelet-derived growth factor receptor alpha (PDGFRα), which are downregulated during their terminal differentiation into postmitotic oligodendrocytes. The term NG2 cells or NG2 glia is used synonymously with OPCs, regardless of whether the cell differentiates into an OL or continues to exist as an OPC for an extended period of time, as currently evidence is lacking that OPCs comprise cells with dichotomous OL differentiation dynamics.

**Figure 1:**
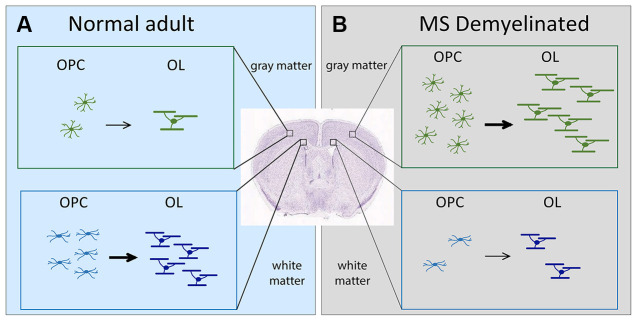
Schematic showing the change in OL dynamics from normal to demyelinated lesion. **(A)** (left, light blue). OPC proliferation and OL differentiation occurs more robustly in the white matter (corpus callosum) than in the gray matter (neocortex) of normal adult brain. **(B)** (right, gray). In MS, OPC recruitment and OL differentiation occurs more robustly in gray matter lesions compared with white matter lesions. The extent of OPC proliferation and OL differentiation is indicated by the number of cells. Arrows indicate differentiation into myelinating OLs. The thickness of the arrows indicates the extent of differentiation. OLs, oligodendrocytes; OPCs, oligodendrocyte precursor cells; MS, multiple sclerosis.

OPCs arise from discrete germinal zones in the embryo and continue to be generated from the subventricular zone in the mature CNS. Despite the reports that nearly all OPCs are in active cell cycle state in the adult brain (Rivers et al., 2008; Psachoulia et al., 2009; Kang et al., 2010), OPCs are heterogeneous in their proliferative rate. In the normal rodent CNS, OPCs proliferate and differentiate into OLs at a higher rate in the white matter than in the gray matter (Hill and Nishiyama, 2014; Boshans et al., 2020; Nishiyama et al., 2021; Figure 1, left). Myelination continues throughout life beyond developmental myelin production and appears to be finely tuned to the local neural network (Monje and Karadottir, 2020; Nishiyama et al., 2021; Pease-Raissi and Chan, 2021).

Damage to myelin occurs in demyelinating diseases such as multiple sclerosis (MS; Lassmann, 2019). Poor remyelination of chronic MS lesions could result from failure of OLs to myelinate axons (Chang et al., 2002) or defects in OPCs (Jäkel et al., 2019; Yeung et al., 2019). Based on the OL dynamics in the normal brain, one would predict that MS lesions in the white matter would be more efficiently remyelinated. Contrary to this prediction, OL dynamics during remyelination in MS is more robust in gray matter lesions (Peterson et al., 2001), despite a higher density of OPCs and OLs in normal appearing white matter than in normal appearing gray matter of MS brains (Strijbis et al., 2017). In this mini review, we will discuss how the regional heterogeneity of the microenvironment surrounding OL lineage cells might contribute to their region-specific behavior in normal and pathological states.

## Regional Heterogeneity of OL Lineage Cells

It has been debated as to whether OPCs represent a heterogeneous cell population (Dimou and Simons, 2017; Foerster et al., 2019; Boshans et al., 2020). Unlike neurons which can be classified into functionally and transcriptionally distinct cell types, OPCs exhibit a spectrum of phenotypes that varies with the age and anatomical location. Below are examples of the phenotypic variability among OPCs and an emerging hypothesis that these differences represent different cell and functional states rather than transcriptionally distinct subtypes.

### OPC Distribution Does Not Always Parallel OL Distribution

While OPCs are distributed throughout the CNS, their density is higher in white matter than in gray matter (Dawson et al., 2000, 2003; Terai et al., 2003). One determinant of OPC density could be the necessity to generate OLs. Consistently, OPC density is low in CNS regions where unmyelinated axons predominate such as the molecular layer of the cerebellar cortex (Givogri et al., 2002; Sun et al., 2018). However, in other regions the distribution of OPCs does not match that of OLs. For example, in the neocortex OPC are distributed uniformly throughout all cortical layers, whereas myelinating OLs are more abundant in deeper cortical layers (Tomassy et al., 2014). Curiously, OPCs exist in relatively high density in some circumventricular organs (Terai et al., 2003; Zilkha-Falb et al., 2020), where capillaries are fenestrated and their density is high (Gross et al., 1986), suggesting a possible non-progenitor role for OPCs. Indeed, OPCs are involved in feeding and body weight control by modulating leptin-sensing of leptin receptor-expressing neurons in the arcuate nucleus that extend their dendrites into the adjacent median eminence (Djogo et al., 2016).

### Higher Rate of OPC Proliferation and OL Differentiation in White Matter

OPCs in white matter proliferate and generate OLs at a greater rate than those in the gray matter (Figure 1, left; Dawson et al., 2003; Dimou et al., 2008; Psachoulia et al., 2009; Kang et al., 2010; Zhu et al., 2011; Young et al., 2013). In slice culture, OPCs in the white matter of the developing corpus callosum and cerebellum proliferate more robustly in response to exogenous PDGF AA than those in the neocortex and cerebellar cortex, despite similar levels of PDGFRα (Hill et al., 2013). When OPCs are isolated from adult gray and white matter and transplanted into gray and white matter of recipient adult mice (Vigano et al., 2013), those from the white matter but not gray matter differentiate robustly into OLs in the host gray matter, whereas those from both gray and white matter differentiate into OLs in the host white matter, suggesting both intrinsic and environmental influences. In slice culture, homo- and heterotopic transplantation of 300-μm^3^ explants or isolated explant cultures indicated that the proliferative response of OPCs to PDGF AA is determined by signals within the 300-μm^3^ microenvironment around the OPCs (Hill et al., 2013). A recent follow-up study demonstrated that neuropilin-1 (Nrp1), a co-receptor for PDGFRα, is expressed by microglia adjacent to OPCs in white but not gray matter and trans-activates PDGFRα on OPCs (Sherafat et al., 2021), providing an example of a regional microglial difference affecting OPC dynamics (see section “Regional Heterogeneity of Microglia Affects OL Lineage Cells” below).

### OPCs Are Transcriptionally Homogeneous

Single-cell RNA-sequencing (scRNA-seq) has revealed multiple subtypes of OLs, but OPCs are more transcriptionally homogeneous and fail to cluster into transcriptionally distinct subtypes (Zeisel et al., 2015; Marques et al., 2016; Tasic et al., 2016). In scRNA-seq of OPCs from different ages, the OPC cluster that expresses genes involved in mitosis segregates from the other two clusters found, which express similar transcripts but appear in postnatal day 7 (P7) and juvenile/adult CNS, respectively (Marques et al., 2018). These clusters are not sufficiently distinct to allow classification of OPCs into distinct subpopulations but may represent different cell states described in the next section.

### Different Cellular and Functional States of OPCs

OPCs in gray and white matter exhibit different electrophysiological properties shaped by the density of voltage-dependent Na^+^ and K^+^ channels (Na_v_ and K_v_, respectively; Chittajallu et al., 2004; Clarke et al., 2012; Larson et al., 2016). A recent comprehensive analysis of Na_v_, K_v_, and glutamate receptor expression and membrane properties of individual OPCs in the cortex and corpus callosum at different ages revealed unique patterns as a function of age and location (Spitzer et al., 2019). When these data are projected against other cellular properties and transcriptional profiles, some correlations emerge. For example, proliferative OPCs have a higher Na_v_ density, and NMDAR expression coincides temporally with OL differentiation. From these analyses, Karadottir and colleagues propose that OPCs can exist in several different states that include: (1) naive state with no voltage-dependent ion channel expression; (2) proliferative state with high Na_v_ density and a transcriptomic profile of mitotic cells; (3) state primed for OL differentiation with high NMDAR density and higher transcripts for OL and myelin genes; and (4) quiescent state with reduced Na_v_ and NMDAR density (Kamen et al., 2021). These states are likely to be affected by their local environment. It is currently not known what specific signals drive them to transition from one state to another, the level of plasticity and convertibility among different states, and how the different OPCs states in turn influence the local neural network.

## Heterogeneity of The Pericellular Environment Around OL Lineage Cells

Many excellent reviews have been published on how neuronal activity influences OL lineage cell behavior. Here, we will discuss how non-neuronal elements might affect the behavior of OL lineage cells. Recent studies have revealed regional heterogeneity of astrocytes and microglia. Furthermore, the vascular supply and vascular mural cell function are also likely to contribute to the different behavior of OL lineage cells in the cortex and corpus callosum.

### Regional Heterogeneity of Astrocytes Affects OL Lineage Cells

Astrocytes are involved in a variety of functions from homeostasis of extracellular K^+^ and glutamate to the regulation of synapses (Khakh and Sofroniew, 2015), and they influence OL development and function in a variety of ways (Barnett and Linington, 2013; Lundgaard et al., 2014; Tognatta et al., 2020). In response to a variety of insults, astrocytes become reactive and upregulate different sets of transcripts depending on the nature of the insults (Escartin et al., 2021). Transcriptional profiling showed that cortical astrocytes are enriched in genes required for cholesterol metabolism, and gray matter astrocytes support *in vitro* myelination more than 2-fold compared with white matter astrocytes (Werkman et al., 2021). Cholesterol is critical for myelination (Saher et al., 2005). Since cholesterol does not cross the blood-brain barrier, cholesterol must be made available to OLs autonomously by biosynthesis in OLs and by horizontal transfer from astrocytes (Camargo et al., 2017). In mice with experimental autoimmune encephalitis (EAE), astrocytes express lower levels of transcripts necessary for cholesterol biosynthesis (Itoh et al., 2018), and astrocytes from MS brains have lower expression of cholesterol biosynthesis genes.

**Figure 2:**
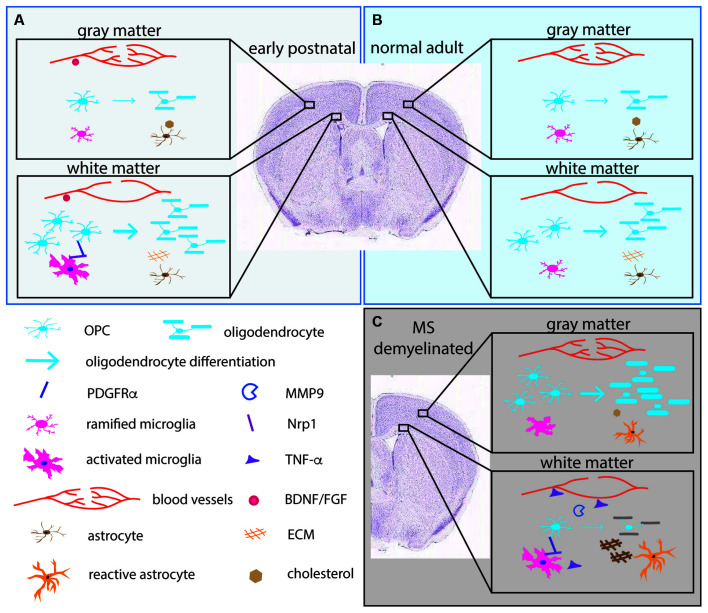
The pericellular microenvironment affecting OPC dynamics under different conditions. **(A)** (upper left box, pale blue). Early postnatal brain **(B)** (upper right box, light blue). Normal adult brain **(C)** (lower right box, gray). Demyelinated lesions in MS brain or experimentally demyelinated lesions. OL lineage cells and their protein products (PDGFRα, MMP9) are depicted in shades of blue. Microglia and their products (Nrp1, TNF-α) are indicated in shades of pink. Astrocytes and their products (cholesterol, ECM) are indicated in orange/brown. Blood vessels and their products (BDNF, FGF) are in red. Arrows indicate differentiation into myelinating OLs. The thickness of the arrows indicates the extent of differentiation. The thick myelin in the gray matter in **(C)** represents successful remyelination. The gray myelin in the white matter in **(C)** represents failure to remyelinate in white matter. The depiction is meant to illustrate examples of the differences and is not meant to be a comprehensive illustration. ECM, extracellular matrix; FGF, fibroblast growth factor; BDNF, brain-derived neurotrophic factor; PDGFRα, platelet-derived growth factor receptor alpha; MMP9, matrix metalloproteinase-9; Nrp1, neuropilin-1; TNF-α, tumor necrosis factor alpha.

By contrast, astrocytes from the white matter of normal young adult rats express extracellular matrix (ECM) genes more abundantly than gray matter astrocytes (Werkman et al., 2020; Figure 2). ECM genes enriched in white matter astrocytes include ECM genes that are upregulated in reactive astrocytes, such as CD44 and collagen genes though they are expressed at a lower level in normal white matter (Zamanian et al., 2012). CD44 is a receptor for the large extracellular proteoglycan hyaluronan which inhibits OL maturation (Buser et al., 2012). Thus, astrocytes from adult white matter appear to be less supportive of myelination compared to those from gray matter. By contrast, those in the gray matter appear to metabolically supportive of OLs and myelin production.

### Regional Heterogeneity of Microglia Affects OL Lineage Cells

Microglia and border-associated macrophages make up the tissue macrophages of the CNS (Prinz et al., 2021). Microglia exhibit regional heterogeneity in their morphology and density (Lawson et al., 1990). They are highly plastic cells that switch from a resting homeostatic phenotype to an activated phagocytic state triggered by a variety of insults including MS lesions (Lassmann et al., 2001). In experimentally induced demyelination, activated microglia initially contribute to demyelination (Marzan et al., 2021) but are subsequently replaced by repair-promoting microglia (Lloyd et al., 2019). Microglia not only sense pathology but also play essential roles in normal development and homeostatic processes, such as neurogenesis, synaptic pruning, and regulation of neuronal activity (Butovsky and Weiner, 2018; Thion et al., 2018; Badimon et al., 2020).

Recent studies suggest that there are region-specific interactions between microglia and OL lineage cells. The early postnatal (P1-P8) corpus callosum contains amoeboid microglia, also referred to as the “fountain of microglia”. These are morphologically distinct from the ramified microglia in the cortex and have the transcriptional signature of activated phagocytic cells (Hagemeyer et al., 2017; Figure 2). They are likely to correspond to the phagocytic microglia seen by electron microscopy that were seen to have ingested “spongioblasts”, which are likely to have been OPCs (Ling, 1976). Depletion of microglia with an antagonist to colony stimulating factor 1 receptor (CSF1R) during the first postnatal week reduces the number of OPCs and OLs (Hagemeyer et al., 2017). Another study showed that a similar population of activated microglia in the early postnatal corpus callosum that express CD11c secrete insulin-like growth factor 1 (IGF1) and play a critical role in developmental myelination (Wlodarczyk et al., 2017). Subsequently published two scRNA-seq studies revealed a microglial subtype that exists in the postnatal white matter that is transcriptionally distinct from developing cortical microglia or adult resting/homeostatic microglia (Hammond et al., 2019; Li et al., 2019). These cells have been termed axon-tract-associated microglia (ATM; Hammond et al., 2019) or proliferative-region-associated microglia (PAM; Li et al., 2019). They express CD68 and other newly identified signature genes such as Spp1 and Clec7A.

Independently, we have shown that activated microglia in the postnatal white matter (ATM/PAM) but not ramified microglia in the cortex express neuropilin-1 (Nrp1), a co-receptor for vascular endothelial cell growth factor (VEGF; Pellet-Many et al., 2008) as well as PDGFRα (Ball et al., 2010; Sherafat et al., 2021). After a demyelinating injury, Nrp1 is strongly upregulated on activated microglia, and microglial Nrp1 deletion severely compromises OPC recruitment and subsequent remyelination (Sherafat et al., 2021). Furthermore, exogenous Nrp1 promotes PDGF AA-mediated OPC proliferation, most potently under conditions of limited amounts of PDGF AA, and exogenous Nrp1 increases tyrosine phosphorylation of PDGFRα on OPCs (Sherafat et al., 2021), suggesting that Nrp1 on ATM/PAM activates PDGFRα on adjacent OPCs in trans (Figure 2). While the mechanism that causes the ATM/PAM to adopt an activated phenotype in the early postnatal corpus callosum remains unknown, apoptosis of early-born ventrally derived OPCs (Orduz et al., 2019) or a sudden local drop in OPC density due to their rapid OL differentiation could trigger their appearance. Future studies could be directed toward elucidating the molecular signaling mechanisms that lead to the emergence of ATM/PAMs in the developing white matter in the context of a homeostatic process for maintaining the correct level of myelin and myelinating cells in the CNS. In demyelinated lesions, the tissue-damaging pro-inflammatory effects of activated microglia, including secretion of inflammatory cytokines such as tumor necrosis factor alpha (TNF-α) are offset by the repair-promoting functions of microglia in a finely coordinated fashion (Lloyd et al., 2019; Figure 2).

### Regional Heterogeneity of Brain Microvessels and the Oligovascular Niche

Evidence is accumulating for a functional interplay between OL lineage cells and the vasculature (Figure 2). In the developing CNS, OPCs migrate along blood vessels (Tsai et al., 2016), and factors from endothelial cells such as fibroblast growth factor 2 (FGF2) and brain-derived neurotrophic factor (BDNF) promote OPC survival and proliferation (Arai and Lo, 2009). Reciprocally, under pathological conditions such as inflammation, OPCs secrete matrix metalloproteinase-9 (MMP9), which increases blood-brain barrier permeability and in turn trigger further tissue damage such as demyelination (Seo et al., 2013). This has led to the concept of the “oligo-vascular niche”, which extends the original concept of the “neurovascular niche” to include OL lineage cells. Recent findings suggest that there is reciprocal signaling between OPCs and vascular mural cells (Miyamoto et al., 2014; Kishida et al., 2019), and that neuronal activity affects myelination through altered cerebral blood flow (Swire et al., 2019).

In rat, the capillary density of the cortex is 3–5 times greater than that of the white matter and correlates with glucose utilization (Borowsky and Collins, 1989; Figure 2). As pial capillaries perforate the cortex during embryonic development, they bring with them meningeal mesenchymal tissue which remains in the adult as the Virchow-Robin complex (Marin-Padilla, 2012). These regional differences in the capillary network could contribute to the regional differences in the functions of OL lineage cells. We recently reported that 94% of OPC processes have close contact with blood vessels in the neocortex of adult mice, and conversely 92% of the vascular segments are contacted by OPC processes (Pfeiffer et al., 2021), suggesting a significant functional interaction between OPCs and vascular mural cells. It remains to be determined whether there are regional differences in the extent of physical relation between OPCs and the vasculature and the functional significance of the OPC-vascular contacts.

## Regional Heterogeneity in MS Lesions

Since the original description of patients with MS by Jean-Martin Charcot^1^ in 1868 and an earlier drawing by Sir Robert Carswell in 1838^2^, MS has been known as a disease that affects the white matter, causing demyelination and subsequent scarring and atrophy. More recent studies indicate that MS also affects the gray matter (Brownell and Hughes, 1962). Myelin basic protein immunohistochemistry revealed frequent cortical demyelinated lesions in autopsied brains from active (Peterson et al., 2001) and chronic (Albert et al., 2007) MS patients. Cortical lesions are more commonly seen in a subpial location and contain fewer inflammatory cells. Furthermore, the extent of cortical demyelination is significantly greater in patients with secondary progressive disease of long duration and in primary progressive MS, and pathological hallmarks for neuronal degeneration and apoptosis are present in cortical lesions (Peterson et al., 2001). The subpial cortical demyelination occurs specifically in MS and not in other conditions similar inflammatory mediators and oxidative tissue damage are involved (Junker et al., 2020).

Despite the widespread nature of cortical demyelination, remyelination is significantly greater in cortical lesions than in white matter lesions, particularly in chronic lesions (Albert et al., 2007). This is accompanied by a greater abundance of OPCs and OLs in cortical lesions compared to white matter lesions (Albert et al., 2007; Chang et al., 2012; Figure 1). Curiously, the converse is true for normal-appearing tissues in MS brains, where OPCs and OLs are found in greater numbers in normal appearing white matter than in normal appearing gray matter (Strijbis et al., 2017), similar to their distribution in normal rodent brains.

What makes OPCs in the gray matter more competent to respond to demyelinated lesions and promote repair? Some observations suggest inherent differences between OPCs in the cortex and white matter in their ability to respond to inflammatory and recruitment signals. For example, OPCs isolated from rat gray matter are more immature and are less responsive to the inhibitory effects of inflammatory cytokines such as interferon gamma (IFN-γ) and TNF-α on OL differentiation (Lentferink et al., 2018). It is also interesting to note that OPCs that develop later from dorsal germinal zones are recruited more robustly to demyelinated lesions than ventrally derived OPCs (Crawford et al., 2016). A recent scRNA-seq study on OL lineage cells from the white matter of postmortem brain from MS patients revealed a significant reduction in the number of cells with the gene expression profiles of OPCs and intermediate cells that are between OPCs and newly formed premyelinating OLs (Jäkel et al., 2019). Perhaps, gray matter OPCs that have not gone through as many rounds of replication as white matter OPCs suffer less from replicative senescence. A transcriptomic comparison between OL lineage cells in gray and white matter MS lesions may provide some answers to this.

On the other hand, there is quite strong evidence that suggests that environmental factors play a major role in shaping OL lineage cell dynamics that differ between gray and white matter MS lesions. White matter MS lesions contain more extensive reactive astrogliosis and more abundant inhibitors of myelination such CD44 and hyaluronan, many of which are produced by astrocytes (Chang et al., 2012). In addition, inflammatory cells including leukocytes and microglia are more prevalent in white matter lesions and provide an importance source of ECM proteins that hinder myelin repair (Ghorbani and Yong, 2021). After acute demyelination in the white matter, the ability of activated microglia and macrophages to successfully undergo efflux of ingested cholesterol influences the efficiency of subsequent remyelination, and this declines with age (Cantuti-Castelvetri et al., 2018). Furthermore, sterol synthesis in phagocytes is necessary to activate LXR signaling to trigger cholesterol efflux and promote resolution of inflammation and subsequent myelin repair (Berghoff et al., 2021).

Intriguingly, the distribution of cortical lesions appears to coincide with the distribution of the vascular supply (Kidd et al., 1999). The meninges have recently been shown to contain a supply of glial progenitor cells that migrate into the CNS parenchyma and contribute to oligodendrogliogenesis (Dang et al., 2019). These progenitor cells from the meninges could be a more efficient source of remyelinating OLs for subpial cortical MS lesions than the neural progenitor cells in the subventricular zone. Future studies may be directed toward understanding how the meningeal inflammatory infiltrates in cortical MS lesions affect the ability of meningeal progenitor cells to migrate into the cortex and contribute to myelin repair. The greater abundance of blood vessels in the cortex and their associated Virchow-Robin space could play an important pathophysiological role in the dynamics of OL lineage cells in the demyelinated cortex.

## Concluding Remarks

Despite the phenotypic variation of OPCs in different neuroanatomical regions, there has so far been no definitive evidence that OPCs can be segregated into distinct subtypes based on permanent changes in gene expression. Rather, OPCs weave in and out of different cell and functional states depending on their physiological context and microenvironment. The specific signals that cause OPCs to alter their functional states have yet to be elucidated. All the elements surrounding OPCs, including neurons, non-neuronal cells, vascular cells, and the ECM exert specific effects on OPC dynamics, and the nature of these signals can change dramatically under certain pathological conditions.

## Author Contributions

AS wrote a draft of the manuscript. FP wrote a draft of the manuscript on the vasculature (“Regional Heterogeneity in MS Lesions” section). AN edited the manuscript. All authors contributed to the article and approved the submitted version.

## Conflict of Interest

The authors declare that the research was conducted in the absence of any commercial or financial relationships that could be construed as a potential conflict of interest.

## Publisher’s Note

All claims expressed in this article are solely those of the authors and do not necessarily represent those of their affiliated organizations, or those of the publisher, the editors and the reviewers. Any product that may be evaluated in this article, or claim that may be made by its manufacturer, is not guaranteed or endorsed by the publisher.

## Footnotes

^1^Charcot JM. Leçons de 1868; Manuscrits des leçns de JM Charcot.: Fonds numérisé Charcot. Bibliothèque de l’Université Pierre & Marie Curie (http://jubilotheque.upmc.fr/subset.html?name=collections&id=charcot).

^2^Pathological Anatomy Illustrations of the Elementary Forms of Disease, London: 1838. https://www.gla.ac.uk/myglasgow/library/files/special/exhibns/month/oct2003.html.
